# Supplementary figures and images for: Differential Proteomics Based on TMT and PRM Reveal the Resistance Response of Bambusa pervariabilis × Dendrocalamopisis grandis Induced by AP-Toxin
Source: Metabolites. 2019 Aug 10;9(8):166. doi: 10.3390/metabo9080166 (PMC6724075; doi:10.3390/metabo9080166)

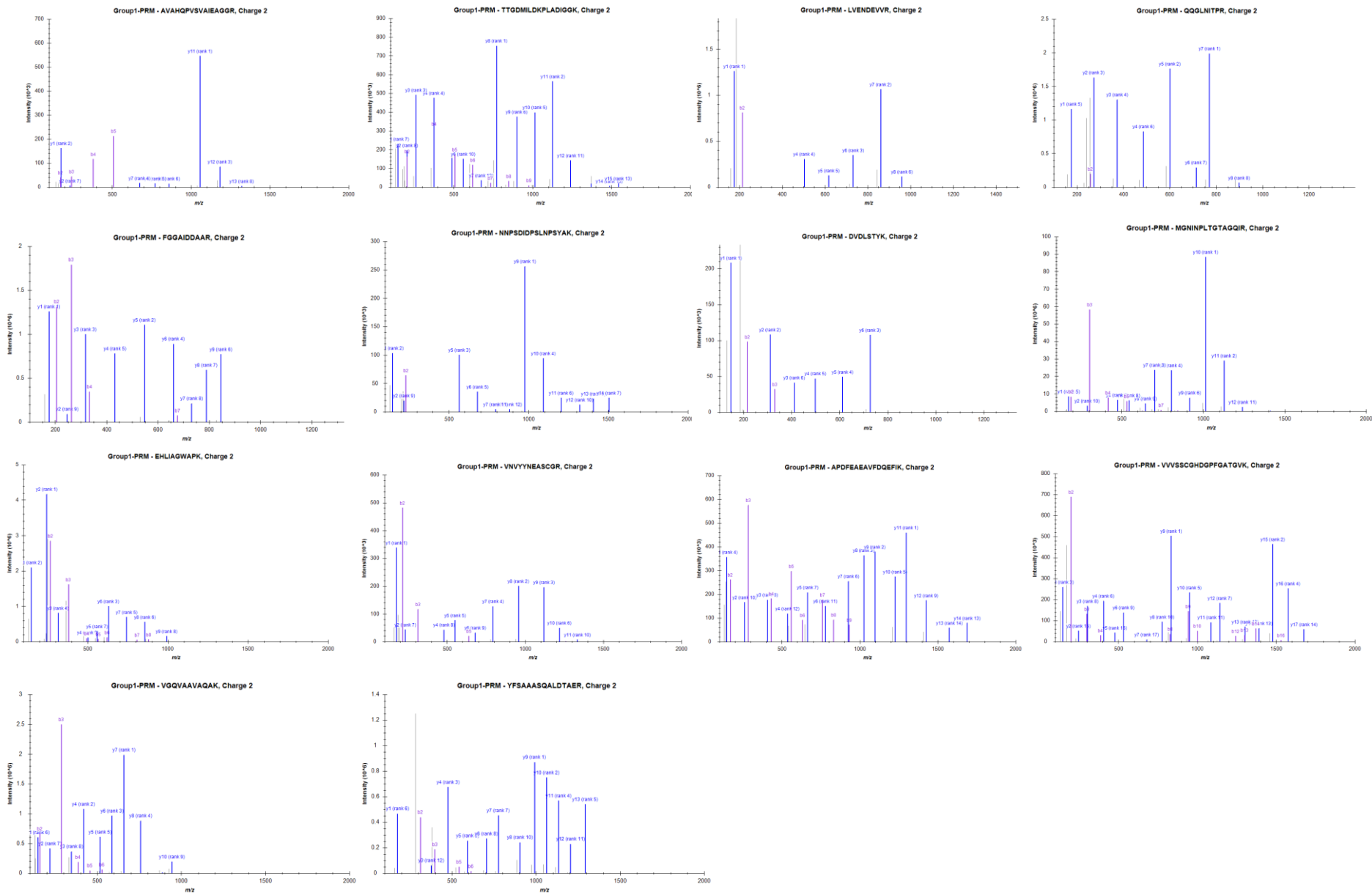

Figure S1. Secondary spectrum of candidate peptides

Supplement: Supplementary file 1 [file metabolites-09-00166-s001.zip › Supplementary files/Figure S1.pdf]
